# On the protective effect of omega-3 against propionic acid-induced neurotoxicity in rat pups

**DOI:** 10.1186/1476-511X-10-142

**Published:** 2011-08-19

**Authors:** Afaf K El-Ansary, Sooad K Al-Daihan, Amina R El-Gezeery

**Affiliations:** 1 Biochemistry Department, Science College, King Saud University, P.O box 22452, zip code 11495, Riydh, Saudi Arabia

**Keywords:** Propionic acid, Omega-3, Neurotransmitters, Caspase-3, Cytokines, Phospholipids, Autism

## Abstract

**Backgrounds:**

The investigation of the environmental contribution for developmental neurotoxicity is very important. Many environmental chemical exposures are now thought to contribute to the development of neurological disorders, especially in children. Results from animal studies may guide investigations of human populations toward identifying environmental contaminants and drugs that produce or protect from neurotoxicity and may help in the treatment of neurodevelopmental disorders.

**Objective:**

To study the protective effects of omega-3 polyunsaturated fatty acid on brain intoxication induced by propionic acid (PPA) in rats.

**Methods:**

24 young male Western Albino rats were enrolled in the present study. They were grouped into three equal groups; oral buffered PPA-treated group given a nuerotoxic dose of 250 mg/Kg body weight/day for 3 days; omega-3 - protected group given a dose of 100 mg/kg body weight/day omega-3 orally daily for 5 days followed by PPA for 3 days, and a third group as control given only phosphate buffered saline. Tumor necrosis factor-α, caspase-3, interlukin-6, gamma amino-buteric acid (GABA), serotonin, dopamine and phospholipids were then assayed in the rats brain's tissue of different groups.

**Results:**

The obtained data showed that PPA caused multiple signs of brain toxicity as measured by depletion of gamaaminobyteric acid (GABA), serotonin (5HT) and dopamine (DA) as three important neurotransmitters that reflect brain function. A high significant increase of interlukin-6 (Il-6), tumor necrosis factor-α (TNF-α) as excellent markers of proinflammation and caspase-3 as a proapotic marker were remarkably elevated in the intoxicated group of rats. Moreover, brain phospholipid profile was impaired in PPA-treated young rats recording lower levels of phosphatidylethanolamine (PE), phosphatidylserine (PS) and phosphatidylcholine (PC).

**Conclusions:**

Omega-3 fatty acids showed a protective effects on PPA - induced changes in rats as there was a remarkable amelioration of most of the measured parameters (i.e. higher GABA, 5HT, DA, PE, PS and PC) and lower Il-6, TNF-α and caspase-3.

## Introduction

There are several reasons to study and test environmental exposures for developmental neurotoxicity: [1] Five thousand new chemicals are produced each year, and according to estimates by the Environmental Protection Agency, about 25% of them may be neurotoxic, but only 10% of them will ever be tested for neurotoxicity [2]. The developing brain is highly vulnerable and more susceptible to neurotoxic insults than is the adult brain [3]. Environmental chemical exposures are now thought to contribute to the increases in attention deficit hyperactivity disorder, cognitive problems, and autism spectrum disorders that have been documented in the last 5-10 years [4]. Results from animal studies could be helpful in identifying environmental toxins that related to neurodevelopmental disorders as autism [1].

Neurotransmitters play unique trophic roles in brain development. Accordingly, drugs and environmental toxicants that promote or interfere with neurotransmitter function evoke neurodevelopmental abnormalities by disrupting the intensity of neuro trophic actions [2].

The brain phospholipids are exceptionally rich in highly unsaturated fatty acids (HUFAs) [3,4] and in contrast to other bodily tissue, a unique feature of neurons is the smaller amounts of the precursors LA(18:2, n-6) and ALA(18:3, n-3) and the higher amounts of their metabolites: AA(20:4, n-6) and DHA(22:6, n-3) [3-5].

Propionic acid (PPA) is a weak organic acid that can cross the lipid bilayer of neuronal membranes and cause mild, reversible intracellular acidification, which can produce wide spread effects on neurotransmitter release involving glutamate, dopamine, and serotonin, each of which can influence locomotion and other behaviors [6-8].

Dependant mechanism may be important for effects of PPA on behavior or cognition. In addition, PPA is thought to affect mitochondrial fatty acid metabolism by binding to propionyl coenzyme A and by sequestering carnitine [[9,10], El-Ansary A, Nounou H: On the effectiveness of creatine and cyclosporine to protect against propionic acid - induced neurotoxicity, submitted). Autism has been suggested to be a mitochondrial disorder of impaired fatty acid metabolism [11,12]. Recently, Macfabe et al (2011) [13] reported that rats treated with PPA displayed some autistic features as restricted behavioral interest to a specific object among a group of objects, impaired social behavior, and impaired reversal in a T-maze task compared to controls given phosphate buffered saline. Moreover the reactive astrogliosis and activated microglia, indicating an innate neuroinflammatory response in PPA-treated rats.

Despite great efforts are directed towards the development of effective treatment for autism, only few strategies have been offered to autistics thus far. The identification of an environmental risk factor related to autism could help to develop a neuroprotective strategy which could delay the onset of the disease. Several studies now recognize the central role of omega-3 polyunsaturated fatty acids (n-3 PUFAs) in brain health. Docosahexaenoic acid (DHA; 22:6n-3) is the most common n-3 PUFA within phospholipids of brain cell membranes, while eicosapentaenoic acid (EPA; 20:5n-3) is usually found in very limited amounts in the central nervous system (CNS) [14,15]. The main dietary sources of long chain PUFA, namely EPA and DHA, remain fatty fish consumption. Besides their critical role in cell membrane fluidity, PUFAs can be released from the hydrophobic moiety of phospholipids via the enzyme phospholipase A2 (PLA2) from which they can be solicited in several intracellular events [16,17]. Although specific mechanisms of action through which n-3 PUFAs act remain unknown, some pathways have been suggested and studied in vitro as well as in vivo. These include anti-inflammatory [18,19], anti-oxidant [18,20,21] and anti-apoptotic properties [22].

This information initiates our interest to study the neuroprotective effects of omega-3 on PPA-induced neurotoxicity through the measurement of neurotransmitters, cytokines, caspase-3 and phospholipids in control, PPA-treated and omega-3-protected-PPA intoxicated rat pups.

## Materials and methods

### Animals

A total of 24 young male rats weighed about 45-60 (approximately 21 days old) were used in the present study. Rats were randomly allocated to the following groups. Oral buffered PPA-treated group given a nuerotoxic dose of 250 mg/Kg body weight/day for 3 days as, n = 8 [23] (omega-3 - protected group given a dose of 100 mg/kg body weight/day omega-3 orally daily for 5 days followed by PPA for 3 days, n = 8. A third group of 8 rats were used as control given only phosphate buffered saline. All groups were kept at controlled temperature (21 ± 1°C) with *ad libtium *access to food and water. All experiments were performed in accordance with national animal care guidelines and were pre-approved by the faculty ethics committee, King Saud University.

### Tissue preparation

At the end of experiment, rats were anesthetized with carbon dioxide. The brain was removed from the skull and was cut into small pieces. Brain tissues of the three groups of rats were homogenized in 10 times w/v bidistilled water and were kept at -80°C until use.

### Biochemical analyses

#### - Assay of tumor necrosis factor-α

Tumor necrosis factor *-α *was measured using ELISA kit, a product of Hycult Biotech. The antibody reacts with rat natural TNF *-α *and recognizes both membrane and receptor bound. The TNF-*α *trimer interacts with either of the two types of TNF-R leading to receptor cross-linking. One unit of Hycult Biotech Mouse Tumor Necrosis Factor *-α *approximates the bioactivity of 16 units of the human Tumor Necrosis factor *-α *standard prepared by WHO as determined in L929 cytotoxic assay.

#### - Assay of Caspase-3

Caspase-3 was measured using an ELISA kit, a product of Cusabio. The microtiter plate provided in this kit has been pre-coated with an antibody specific to Casp-3. Standards or samples are then added to the appropriate microtiter plate wells with a biotin-conjugated antibody preparation specific for Casp-3 and Avidin conjugated to Horseradish Peroxidase (HRP) is added to each microplate well and incubated. Then a TMB (3,3',5,5' tetramethyl-benzidine) substrate solution is added to each well. Only those wells that contain Casp-3, biotin-conjugated antibody and enzyme-conjugated Avidin will exhibit a change in color. The enzyme-substrate reaction is terminated by the addition of a sulphuric acid solution and the color change is measured spectrophotometrically at a wavelength of 450 nm ± 2 nm. The concentration of Casp-3 in the samples is then determined by comparing the O.D. of the samples to the standard curve.

#### - Assay of Interlukin-6

IL-6 was measured using an ELISA kit, a product of Quantikine. A monoclonal antibody specific for rat IL-6 has been pre-coated onto a microplate. 50 μL of Standard, Control, or sample were placed in separate well. The reagent was mixed by gently tapping the plate frame for 1 minute after being covered with the adhesive strip provided. The plate was incubated for 2 hours at room temperature, any rat IL-6 present is bound by the immobilized antibody. After washing away any unbound substances, an enzyme-linked polyclonal antibody specific for rat IL-6 is added to the wells. Following a wash to remove any unbound antibody-enzyme reagent, 100 μL of Substrate Solution was added to each well, and the plate was incubated for 30 minutes at room temperature. The enzyme reaction yields a blue product that turns yellow when the stop Solution is added. The intensity of the color measured is in proportion to the amount of rat IL-6 bound in the initial step. The sample values are then read off the standard curve.

#### - Assay of Gamma amino-buteric acid (GABA)

Quantitative determination of GABA was done using ELISA immunoassay kit, a product of ALPCO. 300 μL of diluted standards, controls and undiluted samples were placed into the appropriate wells of the extraction Plate. 300 μL of the diluent was added to all wells, Covered with Adhesive foil and shakes for 30 min at RT (20-25°C) on a shaker (600 rpm). Two washing cycles were performed, after which 250 μl elution buffers was placed into the appropriate wells of the extraction plate, covered and shaked and then 100 μl of the extract was used for subsequent derivatization. 10 μL of NaOH was added into all wells followed by 50 μL of the equalizing reagent (fresh prepared before assay) and shaked for 1 min on a shaker (600 rpm). 10 μL of the D-reagent was added into all wells, incubated for 2 hours at (20-25°C) and then 150 μL Q-buffer was added into all wells, incubated for 10 min at RT (20-25°C) on a shaker (approx. 600 rpm). 25 μL of the derivatives then used for the subsequent ELISA.

### Assay of serotonin

Serotonin was measured using an ELISA kit, a product of Immunology Biological Laboratories (IBL). Brain homogenate preparation (derivatization of serotonin to N-acylserotonin) is part of the sample dilution and is achieved by incubation of the respective sample with the Acylation Reagent. The assay procedure follows the basic principle of competitive ELISA whereby there is competition between a biotinylated and a non-biotinylated antigen for a fixed number of antibody binding sites. The amount of biotinylated antigen bound to the antibody is inversely proportional to the N-acylserotonin concentration of the sample. When the system is in equilibrium, the free biotinylated antigen is removed by a washing step and the antibody bound biotinylated antigen is determined by use of anti-biotin alkaline phosphatase as marker and p-nitrophenyl phosphate as substrate. Quantification of unknowns is achieved by comparing the enzymatic activity of unknowns with a response curve prepared by using known standards.

#### -Dopamine assay

Dopamine was extracted by using a cis-diol-specific affinity gel, acylated and then derivatized enzymatically. Quantitavive assay was performed using ELISA kit, a product of Immuno Biological Laboratories (IBL).

#### - Phospholipids Measurement

Briefly, 50 μl of brain homogenate was diluted with 750 μl deionized water followed by 2 ml of methanol and 1 ml of chloroform and was mixed well. The mixture was stirred (Rotary mixture 34526, Snijders) for 15 min and centrifuged for 5 min by 4000 rpm [24].

Phospholipid separation was performed on a Kaneur Maxi Star HPLC system with four solvent lines, a degasser SEDEX 55 evaporating light detector (SEDEX 55 Lichtstreu detector, S.E.D.E.E., France) which was coupled with Apex M625 software (Autochrom, USA). As the nebulizing gaz, N_2 _was used at a flow rate of 4l/min, and a nebulizing temperature of 40°C. The gain was set at 8 and 2.0 bar N_2_.

A 125 × 4.0 mm Si-60 column with 5 μm particle diameter (Lichrosher) was used. The elution program was a linear gradient with 80:19.5:0.5 (V/V) chloroform: methanol: water: ammonia (NH_3_) at 22 min and the column was allowed to equilibrate until the next injection at 27 min. The injection volume was 50 μl.

### Statistical analysis

Computer SPSS program was used and results were expressed as mean ± S.D. and all statistical comparisons were made by means of the One-way ANOVA test between the Control, PPA, omega-3+PPA groups and Dunnett test as multiple comparisons. Reciever operating characteristics analysis was done. Area under the curve (AUC), specificity and sensitivity were calculated.

## Results and discussion

Typically an animal model is unlikely to replicate a human disease [25]. The utility of such models relates to the various types of validity that can be shown to exist for specific models. Evidence from human studies suggests that autism is a condition that may involve an ongoing neuroinflammatory disorder possibly resulting from an increased sensitivity to oxidative stress from a variety of environmental risk factors. The biochemical finding of the present study model supports this hypothesis.

Among the omega-3 PUFAs, docosahexaenoic acid DHA is the most important ω3 with physiological significance for brain function [26,27], especially during prenatal brain development when it is incorporated into nerve growth cones during synaptogenesis [28]. Studies have shown that ω-3 dietary deficiency affect the glutamatergic, dopaminergic and serotoninergic systems [29,30]. Glutamate is the main excitatory neurotransmitter in the mammalian central nervous system (CNS), involved in brain plastic processes, such as learning/memory, brain development and ageing [31]. Hyperactivity and anxiety-like behavior have been associated with ω 3 deficiency in various animal models [32-36].

Table 1 demonstrates elevated levels of caspase3, IL-6 and TNF-α as three biochemical parameters related to neuroinflammatory in PPA-treated rat pups. Increased levels of these parameters could be easily correlated to brain injury induced by PPA. On a molecular level, the pro-inflammatory cytokines, TNF- and IL-6, which are produced by immune cells, display an increased expression in depression, multiple sclerosis and other neurological disorders [37,38].

**Table 1:** Mean ± S.D of the measured parameters in control, PPA-treated and Omega-3-protected groups.

Parameters	Groups	Min.	Max.	Mean ± S.D.	P value
	Control	110.34	125.50	119.37 ± 06.56	
	
**Caspase 3**	PPA	137.97	172.31	154.44 ± 12.14 ^a^	0.000
	
	OMEGA	102.60	126.36	115.16 ± 07.80	

	Control	121.33	140.08	129.03 ± 8.06	
	
**IL6**	PPA	129.34	159.37	143.52 ± 9.50 ^a^	0.001
	
	OMEGA	121.58	133.39	127.30 ± 4.33	

	Control	111.01	125.19	119.39 ± 6.10	
	
**TNF**	PPA	125.73	139.32	131.91 ± 4.38 ^a^	0.000
	
	OMEGA	135.17	146.29	139.01 ± 3.63 ^a^	

	Control	95.42	105.03	100.45 ± 4.57	
	
**GABA**	PPA	71.58	90.63	079.09 ± 5.81 ^a^	0.000
	
	OMEGA	100.06	114.78	105.94 ± 5.15	

	Control	5.22	8.32	6.99 ± 1.30	
	
**Serotonin**	PPA	3.10	5.58	4.17 ± 0.85 ^a^	0.000
	
	OMEGA	6.02	9.00	7.01 ± 1.06	

	Control	15.37	19.98	17.91 ± 1.91	
	
**Dopamine**	PPA	6.88	17.63	12.56 ± 3.36 ^a^	0.007
	
	OMEGA	14.14	19.42	16.44 ± 1.83	

	Control	0.13	0.19	0.17 ± 0.03	
	
**PE**	PPA	0.05	0.11	0.09 ± 0.02 ^a^	0.000
	
	OMEGA	0.10	0.16	0.13 ± 0.02 ^a^	

	Control	0.28	0.35	0.31 ± 0.03	
	
**PS**	PPA	0.06	0.18	0.12 ± 0.05 ^a^	0.000
	
	OMEGA	0.22	0.31	0.27 ± 0.03	

	Control	3.82	4.82	4.46 ± 0.46	
	
**PC**	PPA	1.05	2.74	1.97 ± 0.52 ^a^	0.000
	
	OMEGA	2.81	3.86	3.39 ± 0.32 ^a^	

• Significant levels between the three groups are illustrated as superscripts letters when P < 0.05

Table 1 also demonstrates the anti-apoptic effect of omega-3 against the neurotoxic effect of PPA. Caspase 3 was significantly lower in protected animals compared to unprotected PPA-treated rats. This are in good agreement with the previous work of Suphioglu et al (2010) [39] which reported a direct link between DHA treatment and inhibition of apoptosis in M17 cells where more than a 66% reduction in active caspase-3 protein levels was detected in cells treated with 20 μg/ml DHA, compared with the untreated cells. Investigations into the mechanism of apoptotic cell death indicate that the phosphatidylinositol 3-kinase/Akt signalling is a critical pathway in this process and DHA acts in this pathway. In cultured mouse neuroblastoma (Neuro 2A) cells, DHA increased phosophatidyl serine levels resulting in translocation and phosphorylation of Akt at Thr-308, that suppressed activation of caspase-3 [40].

**Figure 1:**
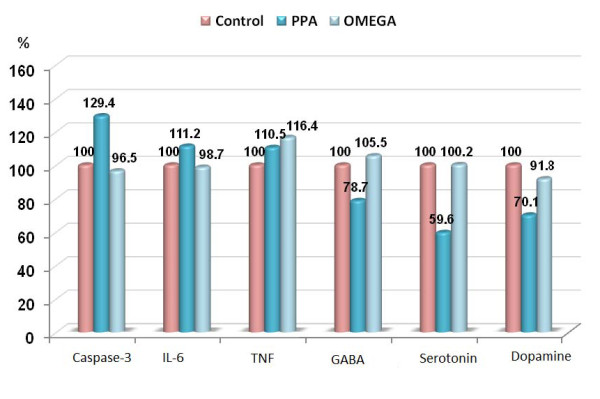
**Percentage change of caspas-3, IL6, TNF-α, GABA, serotonin and dopamine in PPA-treated and omega-3 protected groups compared to control**.

The anti-inflammatory effect of omega-3 reported in the present study and demonstrated in table 1 and figure 1 as significant reduction in the levels of IL-6 and TNF-α induced by PPA could be supported through considering the previous reports of Song and Zhao (2007) [41] on the use of omega-3 in treating depression. Omega-3 fatty acids influence neurogenesis via their anti-inflammatory effect [42]. The mechanisms include that omega-3 fatty acids decrease the production of pro-inflammatory cytokines, such as TNF-α and IL-1β [43] which have been implicated in the mechanism that benefits neurogenesis and cell fate [44].

The remarkable induction of GABA, 5HT and dopamine in rat brain treated with omega-3 as a neuroprotective supplement against PPA neurotoxicity, could find a support through considering the work of Song et al (2009)[43] and Taepavarapruk and Song (2010) [45] which recorded a neurotransmitter regulating and neuroprotective effects of omega-3. They declared that omega-3 neurotransmitter regulatory effect is through the modulation of the quaternary structure of membrane proteins and membrane fluidity, which may benefit the binding of 5-HT [42]. 5-HT, in turn, stimulates neurogenesis in both vertebrate and invertebrate species brain cells [46,47]. Moreover, they reported that, n-3 fatty acids up-regulate levels and expressions of neurotrophins, and its receptors [43,48,49].

As a component of membrane phospholipids, it is documented that the percentage of omega-3 influences the physicochemical properties of the membrane, and thus the function of a variety of membrane-bound proteins, including dopaminergic, GABAergic, and cholinergic receptors *in vitro *[15,50,51] This could support the significant increase in neurotransmitters in omeg-3 protected brains. Ahmada et al (2008) [52] clearly demonstrate lower numbers of dopamine neurons in rats fed a diet deficient in *n*-3 PUFAs, a treatment that has been previously shown to reduce brain phospholipids DHA content by roughly 45%.

**Figure 2:**
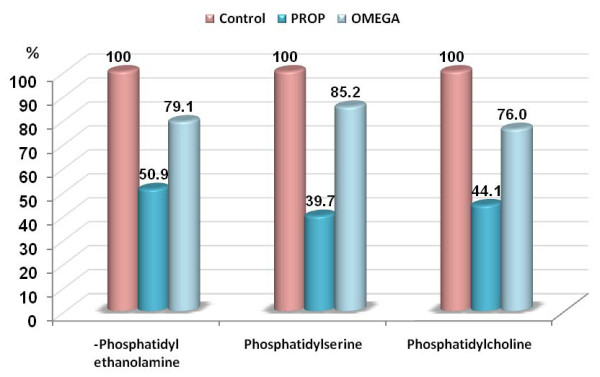
**Percentage change of phosphatidylethanolamine, phosphatidylserine and phosphatidylcholine levels in PPA-treated and omega-3 protected groups compared to control**.

The significant decrease in PE, PS and PC reported in the present study in PPA-treated rats (table 1 and figure 2) could be easily related to brain injury of treated pups. Although the potential key role that phospholipids may have in neurological disorders is not fully understood, multiple lines of evidence to date implicate the lipid environment in the behavior of neurotransmitter systems. Decreased phospholipid PUFAs have been demonstrated in both brain and peripheral membranes in schizophrenic patients, which is consistent with the hypothesis of myelin-related dysfunction in schizophrenia. Membrane defects, such as those induced by decreased PUFAs in phospholipids, can significantly alter a broad range of membrane functions. In addition to increased oxidative stress, altered immune function may also be responsible for increased phospholipase activities. This association is particularly relevant in relation to phospholipids/PUFA, as AA can be converted to a variety of biologically active compounds, such as eicosanoids, which serve as potent messengers in regulating the inflammatory response, as well as endocannabinoids, which may affect brain psychopathology. Direct evidence of immune changes in some patients with autism has come to light, particularly in the activities of several cytokines known to be altered in autoimmune dysfunction. Moreover PUFA are known to regulate neurodevelopment, neurotransmitter homeostasis, PE signaling.

**Figure 3:**
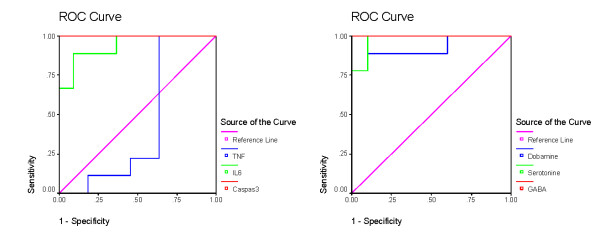
**ROC Curve of caspas3, IL6, TNF, GABA, serotonin and dopamine in PPA group**.

**Figure 4:**
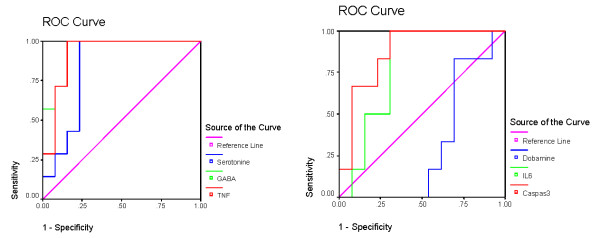
**ROC Curve of caspas3, IL6, TNF, GABA, serotonin and dopamine in omega-protected group**.

**Figure 5:**
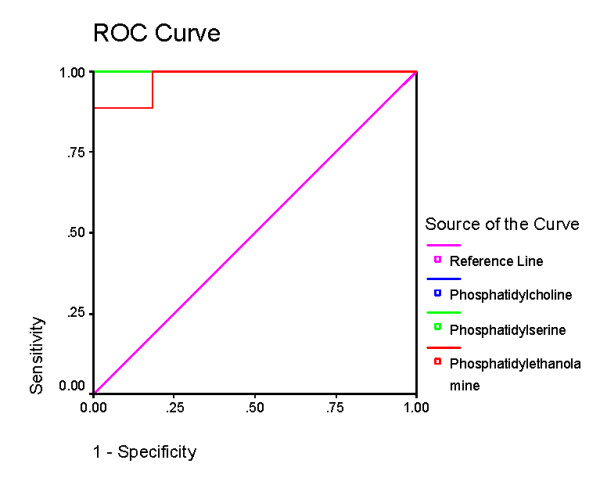
**ROC of phospholipids in PPA-treated group**.

**Figure 6:**
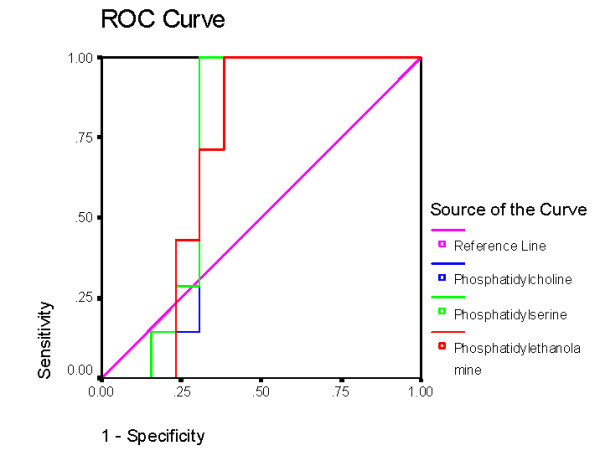
**ROC of phospholipids in omega-3 protected groups**.

Table 2 and figures 3, 4, 5 and 6 demonstrate the ROC analysis of the measured parameters in PPA-treated and omega-protected rats. It could be easily noted that all the measured parameters show satisfactory values of sensitivity and specificity in both groups. This could help to suggest that the investigated parameters could ascertain the neurotoxicity effect of PPA and the effectiveness of omega-3 in ameliorating the toxic effect of PPA.

**Table 2:** ROC analysis of the measured parameters showing, AUC, best cutoff, Sensitivity and specificity values.

Group	Parameter	Area under the curve	Best Cutoff value	Sensitivity%	Specificity%
**PPA**	Caspase3	1.000	132.17	100.0%	100.0%
	
	IL6	0.939	133.59	88.9%	90.9%
	
	TNF	0.434	125.49	100.0%	36.4%
	
	GABA	1.000	93.02	100.0%	100.0%
	
	5HT	0.978	5.80	100.0%	90.0%
	
	DOPA	0.922	14.96	88.9%	90.0%

**OMEGA**	Caspase3	0.872	132.17	100.0%	69.2%
	
	IL6	0.782	133.59	100.0%	69.2%
	
	TNF	0.923	134.50	100.0%	84.6%
	
	GABA	0.945	98.94	100.0%	84.6%
	
	5 HT	0.835	5.80	100.0%	76.9%
	
	DOPA	0.308	17.41	83.3%	30.8%

**PPA**	PE	0.980	0.1184	100.0%	92.%
	
	PS	1.000	0.1994	100.0%	100.0%
	
	PC	1.000	2.7743	100.0%	100.0%

**OMEGA**	PE	0.703	0.1030	100.0%	61.5%
	
	PS	0.725	0.1994	100.0%	69.2%
	
	PC	0.703	2.7743	100.0%	69.2%

**Figure 7:**
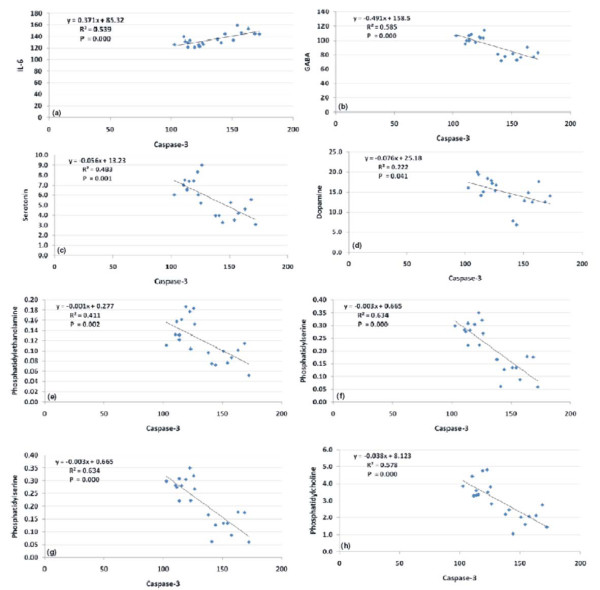
**Pearson correlations between the measured parameters**.

The Pearson's positive correlations seen in table 3 and figure 7 between the measured phospholipids and neurotransmitters (5HT, GABA and DA), and the negative correlations observed between phospholipids and Il-6 and caspase-3 could be explained on the basis that PPA may modulate immune function by stimulating the release of proinflammatory cytokines as TNF-α and IL-6. This immune system modulation or autoimmune response could be directed against lipid membrane components, such as phospholipids (PE, PS and PC). Disruption of brain cells membrane could easily lead to increased efflux of neurotransmitters.

**Table 3:** Pearson correlation test, demonstrating positive and negative correlations between all the measured parameters.

Parameters	R (Person Correlation)	Sig.	
Caspase-3 ~ IL-6	0.734	0.000	P^a^

Caspase-3 ~ GABA	-0.765	0.000	N^b^

Caspase-3 ~ Serotonin	-0.695	0.001	N^b^

Caspase-3 ~ Dopamine	-0.472	0.041	N^b^

Caspase-3 ~ Phosphatidylethanolamine	-0.641	0.002	N^b^

Caspase-3 ~ Phosphatidylserine	-0.797	0.000	N^b^

Caspase-3 ~ Phosphatidylcholine	-0.761	0.000	N^b^

IL-6 ~ GABA	-0.683	0.001	N^b^

IL-6 ~ Serotonin	-0.641	0.002	N^b^

IL-6 ~ Phosphatidylethanolamine	-0.585	0.007	N^b^

IL-6 ~ Phosphatidylserine	-0.576	0.008	N^b^

IL-6 ~ Phosphatidylcholine	-0.690	0.001	N^b^

GABA ~ Serotonin	0.822	0.000	P^a^

GABA ~ Dopamine	0.661	0.002	P^a^

GABA ~ Phosphatidylethanolamine	0.737	0.000	P^a^

GABA ~ Phosphatidylserine	0.839	0.000	P^a^

GABA ~ Phosphatidylcholine	0.703	0.001	P^a^

Serotonin ~ Dopamine	0.657	0.002	P^a^

Serotonin ~ Phosphatidylethanolamine	0.829	0.000	P^a^

Serotonin ~ Phosphatidylserine	0.818	0.000	P^a^

Serotonin ~ Phosphatidylcholine	0.776	0.000	P^a^

Dopamine ~ Phosphatidylethanolamine	0.613	0.005	P^a^

Dopamine ~ Phosphatidylserine	0.679	0.001	P^a^

Dopamine ~ Phosphatidylcholine	0.667	0.002	P^a^

Phosphatidylethanolamine ~ Phosphatidylserine	0.879	0.000	P^a^

Phosphatidylethanolamine ~ Phosphatidylcholine	0.820	0.000	P^a^

Phosphatidylserine ~ Phosphatidylcholine	0.853	0.000	P^a^

• ^**a**^_. _Positive Correlation.

• ^**b**^_. _Negative Correlation.

The previously explained mechanism of the protective effect of omega-3 PUSFA could support the remarkable improvement of most of the measured parameters in ω-3-protected rat pups. This could help to suggest that ω-3 supplementation could be used to ameliorate the impairment in neurotransmitters, cytokines previously recorded as autism-related biochemical features. This suggestion could be supported through considering the previous work of Santiago et al (2010) [53] and Ferraz et al (2011) [54] that ω-3 PUFAs supplementation is associated with increases in hippocampal serotonin and promotes beneficial effects on anxiety, cognitive and depressive-like behaviors in rats subjected to a restraint stress protocol.

## Competing interests

The authors declare that they have no competing interests.

## Authors' contributions

AE designed the study and drafted the manuscript. SD Performed the statistical analysis. AG Helped in drafting the manuscript. All authors have read and approved the final manuscript.
